# MHCSeqNet: a deep neural network model for universal MHC binding prediction

**DOI:** 10.1186/s12859-019-2892-4

**Published:** 2019-05-28

**Authors:** Poomarin Phloyphisut, Natapol Pornputtapong, Sira Sriswasdi, Ekapol Chuangsuwanich

**Affiliations:** 1 Department of Computer Engineering, Faculty of Engineering, Chulalongkorn University, 254 Phayathai Road, Pathumwan, Bangkok, 10330 Thailand; 2 Department of Biochemistry and Microbiology, Faculty of Pharmaceutical Sciences, Chulalongkorn University, 254 Phayathai Road, Pathumwan, Bangkok, 10330 Thailand; 3 Vaccine and Therapeutic Protein, the Special Task Force for Activating Research, Faculty of Pharmaceutical Sciences, Chulalongkorn University, Bangkok, Thailand; 4 Computational Molecular Biology Group, Faculty of Medicine, Chulalongkorn University, Bangkok, Thailand; 5 Research Affairs, Faculty of Medicine, Chulalongkorn University, 1873 Rama IV Road, Pathum Wan, Bangkok, 10330 Thailand; 6 The School of Information Science and Technology, Vidyasirimedhi Institute of Science and Technology, Wangchan Valley 555 Moo 1 Payupnai, Wangchan, Rayong, 21210 Thailand

**Keywords:** MHC epitope prediction, Deep learning, Recurrent neural networks

## Abstract

**Background:**

Immunotherapy is an emerging approach in cancer treatment that activates the host immune system to destroy cancer cells expressing unique peptide signatures (neoepitopes). Administrations of cancer-specific neoepitopes in the form of synthetic peptide vaccine have been proven effective in both mouse models and human patients. Because only a tiny fraction of cancer-specific neoepitopes actually elicits immune response, selection of potent, immunogenic neoepitopes remains a challenging step in cancer vaccine development. A basic approach for immunogenicity prediction is based on the premise that effective neoepitope should bind with the Major Histocompatibility Complex (MHC) with high affinity.

**Results:**

In this study, we developed MHCSeqNet, an open-source deep learning model, which not only outperforms state-of-the-art predictors on both MHC binding affinity and MHC ligand peptidome datasets but also exhibits promising generalization to unseen MHC class I alleles. MHCSeqNet employed neural network architectures developed for natural language processing to model amino acid sequence representations of MHC allele and epitope peptide as sentences with amino acids as individual words. This consideration allows MHCSeqNet to accept new MHC alleles as well as peptides of any length.

**Conclusions:**

The improved performance and the flexibility offered by MHCSeqNet should make it a valuable tool for screening effective neoepitopes in cancer vaccine development.

**Electronic supplementary material:**

The online version of this article (10.1186/s12859-019-2892-4) contains supplementary material, which is available to authorized users.

## Background

Immunotherapy is a promising approach in cancer treatment that activates the host immune system to specifically destroy cancer cells, with far fewer adverse effects than chemotherapy or radiotherapy. This is possible because cancer cells produce unique peptide signatures (neoepitopes), some of which are presented on the cancer cells’ outer surface and recognized by T cells [1, 2]. Administrations of vaccines composed of synthetic peptides resembling cancer-specific neoepitopes have been proven to boost T cell activity to destroy cancer cells in both mouse models and human patients [2–4]. Nonetheless, because only a tiny fraction of hundreds of cancer-specific neoepitopes can elicit immune response, selection of immunogenic neoepitopes remains a challenging step in cancer vaccine development.

A basic approach for immunogenicity prediction is based on the fact that the Major Histocompatibility Complex (MHC), also called Human Leukocyte Antigen (HLA) complex, binds to peptide epitopes and presents them on the outer cell surface for recognition by T cells. In other words, a good neoepitope should be able to bind with MHC molecule with high affinity [5]. Current state-of-the-art software tools for peptide-MHC binding affinity prediction achieved high accuracy due to the availability of large-scale training datasets [6, 7] and the application of artificial neural networks [8–10]. Although recent approaches that employed deep learning models [9, 10] have demonstrated considerable performance gains over established tools including NetMHCpan [8], they limited the length of input peptide epitopes (9 amino acids for ConvMHC and 8-15 amino acids for MHCflurry) and supported only specific MHC alleles that the models had been trained for. In contrast, NetMHCPan can make binding affinity prediction for any peptide and even for MHC alleles not present in the training dataset – as long as the alleles’ amino acid sequences are known.

Amongst different fundamental architectures for deep learning, Recurrent Neural Networks (RNNs) have been used to encode time series information in many tasks such as automatic speech recognition [11], natural language processing [12], and bioinformatics [13]. Unlike fully connected feed forward networks, RNNs can better capture temporal relationships by remembering previous inputs. A popular choice for RNNs is the Long Short-Term Memory (LSTM) which alleviates the vanishing and exploding gradient problems presented in normal RNNs. Gated Recurrent Unit (GRU) is a recently developed model which can be considered as a simplified version of the LSTM [14]. Not only are GRUs easier to train than LSTMs, but also they outperform LSTMs in many tasks [15, 16].

In this study, we developed MHCSeqNet, an open-source deep learning model that can predict peptide-MHC binding with high accuracy and with no restriction on the input peptide or MHC allele, as long as its amino acid sequence is known. Our training and testing datasets were derived from the Immune Epitope Database (IEDB) [6] and recent publications [9, 17]. Compared to NetMHCPan [8] and MHCflurry [9] which pre-process amino acid sequences of peptide and MHC allele into fixed-length inputs for the underlying fully connected or convolutional neural networks, the key innovation of our approach lies in the recurrent neural network architecture which can naturally handle variable-length input amino acid sequences. Furthermore, we utilize a context-aware amino acid embedding model instead of a position-specific encoding system based on amino acid substitution matrices that was used by both NetMHCPan and MHCflurry. This allows us to incorporate multiple amino acid sequence datasets to learn better embedding representations.

Our evaluations show that transfer learning from a larger amino acid database can help improve the embeddings, with further improvements possible through the use of additional model fine-tunings. Representing an MHC allele with the embedding of its amino acid sequences] instead of its type name (one-hot representation) also helps the generalization of the model in most cases. MHCSeqNet outperforms NetMHCPan [8] and MHCflurry [9] on both MHC binding affinity and MHC ligand peptidome datasets. The improved performance and the flexibility offered by MHCSeqNet should make it a valuable tool for screening effective neoepitopes in cancer vaccine development.

## Implementation

MHCSeqNet was implemented using Python 3 and the following packages: numpy version 1.14.3, Keras version 2.2.0, tensowflow version 1.6.0, scipy version 1.1.0, and scikit-learn version 0.19.1. Details and source codes can be found on GitHub at https://github.com/cmbcu/MHCSeqNet.

### Architecture overview

**Fig. 1:**
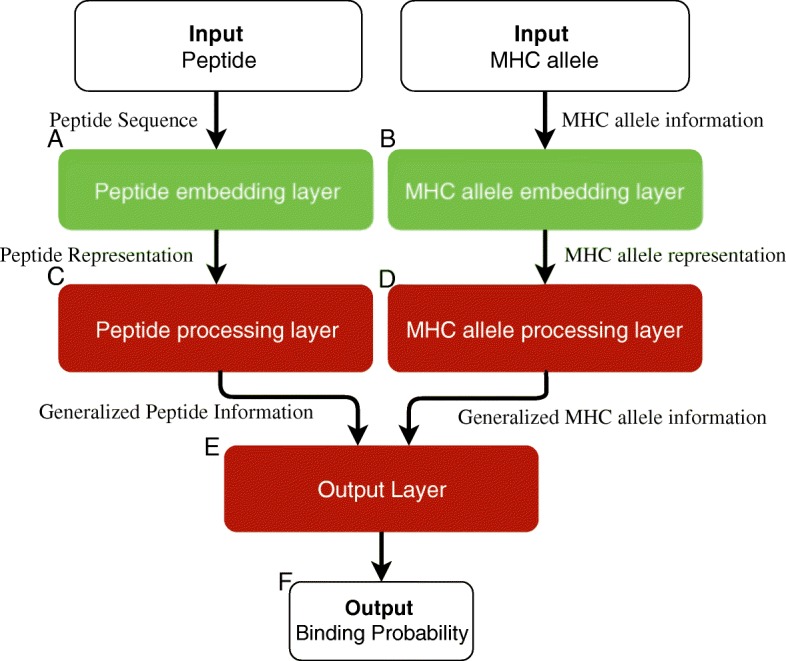
An overview of the MHCSeqNet’s architecture. The model is comprised of three main parts: the peptide sequence processing part (**a** & **c**), the MHC processing part (**b** & **d**), and the main processing part which accepts the processed information from the previous parts (**e**). The entire model is a single deep learning model which can be trained altogether. **f** Our models output binding probability for the given peptide and MHC allele on the scale of 0 to 1, with 1 indicating likely ligand

We trained deep learning models to predict the probability of binding between peptide and MHC allele where a prediction of 0.0 indicates no binding and 1.0 indicates a strong binding. Our models accept two inputs: peptide, in the form of amino acid sequence, and MHC allele, in the form of either amino acid sequence or allele name. Figure 1 shows an overview of our model. The model consists of three main parts, namely the peptide input processing (Fig. 1a and c), the MHC allele input processing (Fig. 1b and d), and the output layer (Fig. 1e). The input processing modules try to learn the best internal representations for peptide and MHC allele. They then pass the processed representations to the output layer which perform the final classification. In the following subsections we will go over each part of the model.

### Peptide embedding layer

We considered two representation models for amino acids. The first is a simple one-hot model where each amino acid is represented by a unit binary vector, e.g. [1,0,0,…] for one amino acid and [0,1,0,…] for another amino acid. The second is a continuous vector representation, called embeddings [18], one of the most successful models in Natural Language Processing (NLP) which can capture the semantic and syntactic relationships between words in a sentence. In our case, a peptide may be considered a sentence and amino acids the individual words. Using embeddings allows us to train the representations on a much larger dataset than the target task (pre-training). The embeddings can then be used or adapted to tasks with smaller datasets.

The Skip-Gram Model [19] was used to train the continuous vectors by treating each set of 1 or 3 consecutive amino acids (1-gram or 3-gram) as a unit. The choice of the 3-gram model, also called ProtVec, was selected according to an earlier study [20]. For each peptide, there are three different 3-gram representations with 0, 1, or 2 amino acid offset from the N-terminus of the peptide. For the tunable parameters, we tested window sizes of 3, 5, or 7 and embedding dimension of 4, 5, and 6 for the 1-gram model. We found that the exact choice of these parameters have little effect on the performance of the model. In the case of the 3-gram model, we fixed the embedding dimension at 100, which was the reported optimal parameter [20].

### Peptide processing layer

GRU was chosen as the peptide processing layer (Fig. 1c) because it is capable of processing sequences with variable lengths and generalizing the relationship between the amino acid representation in peptide sequence. We used one GRU layer. For the 1-Gram amino acid representation, a bi-directional GRU was used. For the ProtVec model (3-gram), three parallel GRU layers (one for each offset) were used. The number of GRU units tested ranged from 32 to 224.

The input to the GRU is the embedded amino acid sequence. We searched for the best amino acid representation by performing 5-fold cross-validation to train and test the entire model for each candidate embedding. We also allowed the model to adapt parameters in the peptide embedding layer via back-propagation. However, the large number of parameters in ProtVec representation caused the model to overfit during the adaptation and ultimately worsened the performance. To overcome this problem, the ProtVec model was trained with the following procedure. First, the model was trained without adaptation until the loss steadied. Then, we enabled adaptation and resumed model training for 1-3 epochs. Finally, adaptation was disabled again, and the model was trained until it stopped improving. This procedure is similar in spirit to other transfer learning methods [21, 22].

### MHC allele embedding layer

We considered representing an MHC allele with its amino acid sequence in order to allow the model to predict binding probabilities for new MHC alleles that were not part of the training dataset, as long as the new allele’s amino acid sequence is known beforehand. We obtained the amino acid sequence of human MHC class I alleles from the Immuno Polymorphism Database (IPD) [23]. Then, we extracted the amino acid sequence portions that correspond to the two alpha helices that participate in the binding with peptide ligand [8], e.g. the residues 50-84 and 140-179 on the structure of HLA-B*35:01 (PDB ID: 1A1N, Additional file 1) [24]. It should be noted that inclusion of amino acid sequences from the beta sheet which was known to participate in ligand binding (e.g., residues 3-37 and 94-126, Additional file 1) into our model worsened the performance. To define the corresponding amino acid residues of the alpha helices in each MHC allele, we used MUSCLE v 3.8.31 [25] with default parameter to create a multiple-sequence alignment of all MHC alleles and then mapped the location of residues 50-84 and 140-179 from HLA-B*35:01 to other alleles.

Each MHC allele is inputted into the MHC allele embedding layer as a sequence of amino acids (Fig. 1b). The MHC allele embedding layer was randomly initialized instead of using pre-trained weights because the alignments can contain gaps which were represented by a special character. For the MHC allele processing layer (Fig. 1d), two different layers were tested, namely the fully connected layer and the GRU. We tuned the models by varying the layer sizes from 50 to 350 neurons for the fully connected layer, and from 8 to 80 for the GRU. Additionally, we implemented a one-hot representation of the MHC allele. This representation ignores the amino acid sequence of MHC allele and therefore does not capture relationship between MHC alleles.

### Output layer

The outputs from both the peptide processing layer and the MHC allele processing layer are then passed through two fully connected layers with rectified linear unit (ReLU) as the activation function (Fig. 1e). A final classification layer employs a sigmoid activation function to obtain the final output in the form of binding probability (Fig. 1f). We varied the layer size of the fully connected layers from 64 to 512 neurons. In order to prevent overfitting, dropouts [26] were also applied to the fully connected and the GRU layers [14]. We tested dropout probabilities of 0, 0.1, 0.2, 0.3, 0.4, and 0.5 and obtained similar performances. In the final configuration, the default dropout probabilities were set at 0.4 for outputs from the fully-connected layers and for inputs to the GRU layers, and 0.3 for recurrent dropout in the GRU layers.

### Neural network training and final model development

We employed a 5-fold cross-validation scheme where the dataset was divided into five partitions of equal size. Entries with the same peptide sequence were also grouped so that all of them are assigned to the same partition. For each fold, four partitions were used for training and the remaining partition for testing. Furthermore, in each fold, we used 20% of the training data for hyperparameter tuning and early stopping of the training process. In our model, architecture weights are shared across all MHC alleles, as suppose to building one model per MHC allele [9]. Adam optimization algorithm was used for the training [27].

The final model was developed by using the representation of peptide and MHC alleles as well as the neural network architectures that achieved highest performances. The best representation for peptide is the 1-Gram model trained on the combination of Swiss-Prot proteins and predicted proteasome-cleaved human peptides as described below. For the best representation of MHC allele, there are two representations which produce similar results. The first candidate is the one-hot amino acid representation coupled with fully connected layers. The second candidate is the one-hot MHC allele representation. Thus, we included both models in the software. We then created a simple ensemble model using the 5 models each trained on one data partition. The final binding probability prediction is defined as the median of the outputs from these 5 models.

### Performance evaluation

The performance of our models were measured using the area under the receiver operating curve (AUC). The AUC were calculated for both the whole test set and for individual MHC alleles. Performance over all 92 MHC alleles were included in the calculation for the whole test set. For individual alleles, we only evaluate 43 MHC alleles that have at least 30 data points in total and at least 5 positive and at least 5 negative data points. AUC across the five folds from cross-validation were averaged. Additionally, we also report the F1 scores. The score is calculated by selecting the threshold that achieves the highest F1 from the receiver operating curve.

We compared the AUCs of our models to those of NetMHCPan 4.0 [8] and MHCflurry version 1.1.0 [9] by using them to make binding affinity predictions on the same test sets as MHCSeqNet. To ensure a fair comparison, MHCflurry was re-trained using our cleaned dataset. We evaluated the impact of MHCflurry’s hyperparameters on its performance by varying the values of two key parameters, namely the number of filters in locally connected layers and the number of layers, to be 8, 16, 32, and 64 (the default values are 8 and 16, respectively), and calculating the corresponding AUCs. Overall, the change in performance is minimal, with standard deviation of AUC among these parameter sets being only 0.0063. Hence, we decided to keep the default hyperparameters for MHCflurry. On the other hand, as the public version of NetMHCPan could not be re-trained, we evaluated its performance as is. In each comparison, only MHC alleles supported by all software tools involved were considered. This restricted the evaluation sets to 41 MHC alleles (Additional file 2). Furthermore, MHCflurry limits the length of input peptides to be between 8 and 15 amino acids while NetMHCPan can make prediction for peptide of any length.

Additionally, we tested all models on an external dataset [17] that consists of MHC class I peptidome from four human individuals whose HLA-A, HLA-B, and HLA-C alleles have been determined. Since a detected ligand in this dataset could be bound to any of the MHC alleles present, we took the maximal predicted binding probability or affinity over the set of MHC alleles in each individual as the prediction of each model. To enable the calculation of AUC here, we also include the negative data from the test set which were not used during the training of our models into this evaluation.

### Prediction for unseen MHC alleles

We evaluated the capability of our sequence-based model to predict peptide-MHC binding for unseen MHC alleles by purposely omitting one MHC allele at a time from the training dataset, retraining the model, and then predicting the binding for peptides contained in the omitted data. This process was repeated for every MHC allele in the dataset and the prediction performance in term of average AUCs over 5 models (derived from 5-fold cross-validation) were recorded.

### Peptide sequence and peptide-MHC binding affinity datasets

To obtain a large amount of amino acid sequences for pre-training the 1-Gram and 3-Gram amino acid representation models described above, we downloaded 468,891 verified protein sequences of all species from Swiss-Prot [28] and also constructed a dataset of 16 million simulated proteasome-cleaved human 9-mer peptides using NetChop [29]. Likely proteasome-cleaved 9-mers were defined as those flanked by cleavage sites with predicted cleavage probability ≥0.5. The 1-Gram and 3-Gram models were then trained on the Swiss-Prot proteins alone, the simulated 9-mers alone, or the combination of the two.

We combined peptide-MHC binding affinity data from IEDB [23] and MHCflurry [9], and selected entries corresponding to human MHC class I molecules (HLA-A, HLA-B, and HLA-C) with peptide ligand lengths between 8 and 15 amino acids. Entries with ambiguous amino acids, namely B, X, J, and Z, or non-specific MHC allele names, such as HLA-A30 or MHC class I, were excluded. Furthermore, as one of our models needs the amino acid sequence of MHC allele as an input, only alleles whose amino acid sequences are present in the Immuno Polymorphism Database [23] were selected.

We chose to disregard all quantitative binding affinity values and used only qualitative binding classifications, namely Positive-High, Positive, Positive-Intermediate, Positive-Low, and Negative, because the binding affinity values were acquired through diverse, non-standardized experimental techniques performed in multiple laboratories. Furthermore, we found that removing low-confidence entries (Positive-Intermediate and Positive-Low) slightly improved the prediction performances. There were also a number of conflicting entries which contained the same MHC allele and peptide ligand but opposite binding classifications. As the majority of these conflicts come from a few published sources (PubMed IDs), we decided to exclude all entries from these sources. For each of the remaining conflicts, we reassigned the binding classification based on the majority vote. If there is an equal number of Positive and Negative entries, all of the associated conflicting entries were excluded from further considerations.

In total, the final cleaned dataset contains 228,348 peptide-MHC entries consisting of 31 HLA-A, 49 HLA-B, and 12 HLA-C alleles. The number of ligands per MHC allele ranges from 41 to 21,480. The cleaned dataset is provided as Additional file 3 and can also be found on MHCSeqNet’s GitHub page.

## Results

### MHC allele representation model

We trained our models using two different MHC allele representations: a one-hot system which conveys no relationship between MHC alleles, and an amino acid sequence-based representation that permits inference across alleles. We also tested two entry layer architectures for processing MHC allele’s amino acid sequences: a fully connected layer and a GRU layer. Evaluations based on AUC showed that using a fully connected layer as the entry layer for the sequence-based models gives slightly better overall performance than using a GRU layer (AUC of 0.9910 and 0.9898, respectively). Compared to sequence-based models, the one-hot MHC allele representation model yielded slightly better overall performance with an AUC of 0.9917 and it outperformed the sequence-based models on almost every MHC allele (41 out of 43 alleles tested, Additional file 2). A closer inspection revealed that the both models achieved similar performance on alleles with large amount of training data. And for MHC alleles with fewer training data points, the performance gap between the one-hot and the sequence-based models tend to be higher. This is likely because the sequence-based model has more parameters and thus requires more data to train.

### Peptide embedding

The one-hot, 1-gram, and 3-gram peptide representations were pre-trained on three different datasets: Swiss-Prot proteins, simulated human proteasome-cleaved 9-mers, and the combination of the two. We also studied the case where no pre-training is done, and the embedding layer was initialized randomly. Overall, the 1-gram model yielded the best performance with an average AUC of 0.991715, followed by the one-hot model with an average AUC of 0.991680. For the 1-gram model, combining the two amino acid sequence datasets slightly improved the performance over using individual dataset or using no pre-training. Rather unexpectedly, regardless of the adaptation method (see Methods) or the dataset tested, the 3-gram model, which has previously been used to analyzed protein structural family [20], consistently performed worse than the other alternatives. We suspected that the large number of parameters in the 3-gram model caused the model to overfit even when adaptation was performed carefully. Nonetheless, significant improvement in AUC for the 3-gram model was achieved with our special adaptation method (from 0.967181 to 0.982490).

### Evaluation using MHC class I binding affinity dataset

**Fig. 2:**
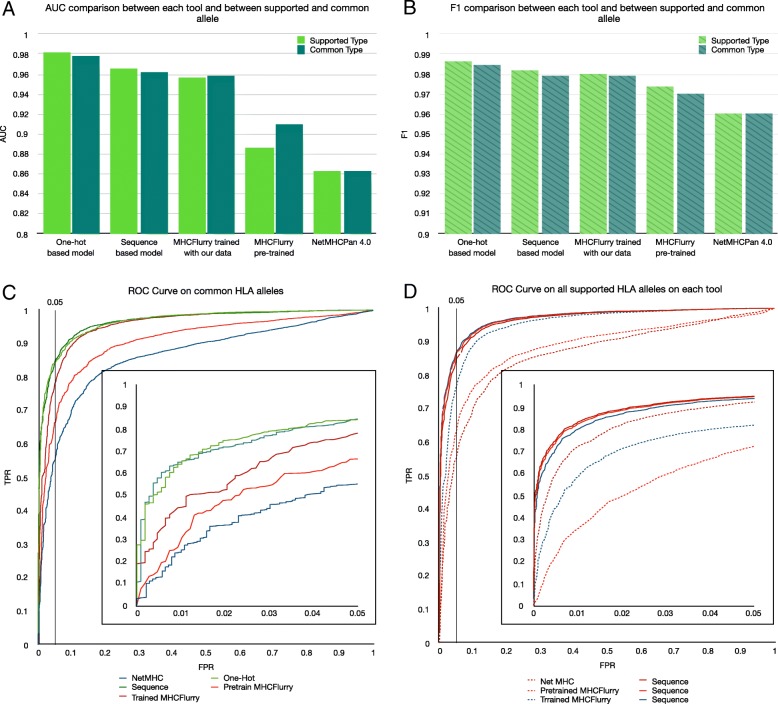
MHCSeqNet achieves the best AUC and F1 scores on MHC class I binding dataset. **a** Bar plots showing the AUC value of each tool when evaluated on the set of MHC alleles it supports (Supported Type) or on the set of MHC alleles supported by all tools (Common Type). **b** Similar bar plots showing F1 values. **c** The ROC plot for all tools when evaluated on the set of MHC alleles supported by all tools. Vertical black line indicates the 5% false discovery rate (FDR). Inset shows the zoomed in ROC plot for the region with ≤5% FDR. **d** Similar ROC plot for the evaluation on MHC alleles supported by individual tools

From above results, the 1-gram model trained on the combination of Swiss-Prot proteins and simulated human proteasome-cleaved 9-mers was selected as the peptide embedding layer. For MHC allele representation, we evaluated both the one-hot and the sequence-based models here. The performance of our models, NetMHCPan version 4.0 [8], MHCflurry version 1.1.0 [9], and the retrained MHCflurry were evaluated on the MHC class I binding affinity dataset using a five-fold cross-validation scheme (see Methods). The dataset was also split so that entries with the same peptide sequences were all assigned to the same cross-validation fold. This revealed that both of our models significantly outperformed NetMHCPan and MHCflurry overall with respect to both AUC and F1 score (Fig. 2). Analysis of 100 bootstrap samples obtained by sampling 80% of entries in the test set with replacement showed that these AUC estimates are highly stable with coefficients of variation smaller than 0.26*%*. Additionally, the binding probabilities predicted by both of our models strongly distinguish between positive and negative ligands. For positive ligands, the vast majority of predicted binding probabilities are close to 1.0 (Additional file 4). For negative ligands, the predicted binding probabilities center around 0.2 with the 75th percentile located at around 0.6.

On individual MHC allele level, our one-hot model achieved the highest AUC than all others on 32 out of 41 alleles that are supported by all tools (Additional file 2). Among 9 alleles where our one-hot model did not yield the best performance, MHCflurry performed better on 8 of them (HLA-A*02:02, HLA-A*02:06, HLA-A*26:01, HLA-A*30:02, HLA-A*33:01, HLA-A*69:01, HLA-B*40:01, and HLA-B*53:01) and NetMHCpan did so on only one allele (HLA-A*68:01). Furthermore, these alleles are mostly alleles with few training data points (8 of these alleles are among the bottom 13 alleles with the least amount of data points). Among NetMHCpan and our sequence-based model – the two approaches that can handle any input MHC allele, our model achieved higher AUC on 32 out of 41 alleles (all alleles except HLA-A*02:02, HLA-A*02:06, HLA-A*23:01, HLA-A*30:02, HLA-A*33:01, HLA-A*68:01, HLA-B*08:01, HLA-B*53:01, and HLA-B*58:01).

### Evaluation using external MHC class I peptidomes

**Fig. 3:**
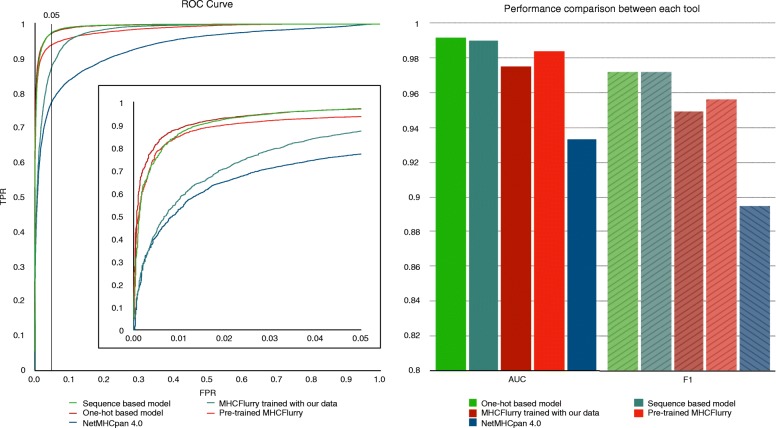
MHCSeqNet achieves the best AUC and F1 scores on MHC class I ligand peptidome dataset. **a** The ROC plot for all tools. Vertical black line indicates the 5% FDR. Inset show the zoomed in ROC plot for the region with ≤5% FDR. **b** Bar plots showing the AUC (bars with solid face colors) and F1 (bars with stripes) scores of each tool

We further evaluated our models, NetMHCPan, and MHCflurry on MHC class I ligand peptidomes [17] which were derived from mass spectrometry analyses of four human individuals whose HLA-A, HLA-B, and HLA-C alleles have been determined (Table 1). This evaluation mimics real use cases where a predicted MHC ligand may bind to any of the MHC class I alleles present in a patient. As it is unclear which MHC allele was bound to each detected peptide, the maximal predicted binding probability or affinity over the set of MHC alleles in each individual was designated as the final prediction for each model. Additionally, to ensure that this test is independent from the evaluation using binding affinity data, all peptidome entries that overlap with our training dataset were removed from consideration. It should be noted that some HLA-C alleles were not supported by NetMHCPan, MHCflurry, and our one-hot model (Table 1), and that the sequence-based model alone could make predictions for these alleles. Again, our models achieved the best overall AUC and F1 scores (Fig. 3).

**Table 1:** Typed MHC alleles of four individuals in the MHC class I ligand peptidome dataset

Sample ID	Mel 12	Mel 15	Mel 16	Mel 8
HLA-A	A*01:01	A*03:01	A*01:01	A*01:01
	-	A*68:01	A*24:02	A*03:01
HLA-B	B*08:01	B*27:05	B*07:02	B*07:02
	-	B*35:03	B*08:01	B*08:01
HLA-C	C*07:01^a^	C*02:02^b^	C*07:01^a^	C*07:01^a^
	-	C*04:01	C*07:02	C*07:01^a^

^a^Alleles not supported by the original MHCflurry

^b^Allele supported by only our sequence-based model

### Prediction for unseen MHC alleles

To evaluate our sequence-based model’s ability to predict peptide-MHC binding probability for new, unseen MHC alleles, we trained the sequence-based model using data from all-but-one alleles and calculated the model’s AUC using data from the omitted allele. To ensure that the AUC estimates are stable, we repeated the training and AUC calculation process with different random initialization five times for each allele and reported the average AUCs. This revealed that the sequence-based MHC allele representation model clearly outperformed the one-hot MHC allele representation model on 47 out of 60 MHC alleles evaluated (Additional file 5) with median AUC of 0.7987 versus 0.6159 and a median AUC difference of 0.12 across all alleles.

## Discussion

### Impact of data cleaning

In addition to removing duplicated entries and entries with ambiguous peptide sequence or MHC allele name as regularly performed in other studies [8, 9], we also examined the impact of conflicting entries (entries with the same peptide sequence and same MHC allele but opposite binding affinity classification) and low-confidence entries (Positive-Intermediate and Positive Low) on the prediction performance. Although conflicting entries constitute less than 5% of the raw dataset, the vast majority of them (15,305 out of 17,914 conflicting entries) involve the same source, which reported HLA-B*27 ligands identified in transgenic mice [30], and should be entirely excluded or at least carefully scrutinized. The remaining conflicts could be resolved by majority voting which slightly improves the prediction performance. We also found that the exclusion of low-confidence entries slightly improved the prediction performances when tested on all entries or on only high-confidence entries.

### Capability to make prediction for unseen MHC alleles

The capability to predict binding affinity of a candidate neoepitope against all MHC alleles presented in a patient is highly desirable because neoepitopes that can bind to multiple MHC alleles are likely to be immunogenic. The sequence-based version of MHCSeqNet not only better predicted ligands for unseen MHC alleles than its one-hot counterpart but also improved over existing tools. Curiously, the sequence-based model did not outperform the one-hot model when tested on a peptidome dataset which contains two HLA-C alleles not present in the training dataset (Table 1). We suspected that this is due to the overall lack of HLA-C data (HLA-C entries constitute only 3% of the final training dataset) for the sequence-based model to learn from. Indeed, the sequence-based model performed worse than the one-hot models on 4 out of 9 HLA-C alleles considered when comparing the the two (Additional file 5). More data on HLA-C epitopes from future experiments should greatly help improve the performance of the sequence-based version of MHCSeqNet.

## Conclusions

MHCSeqNet exhibits performance improvement over existing tools for predicting MHC class I ligands on both binding affinity and peptidome datasets. Furthermore, MHCSeqNet retains the flexibility to make prediction for peptide of any length and for any MHC class I allele with known amino acid sequence by utilizing recurrent neural network architectures to handle amino acid sequences. Thus, MHCSeqNet should contribute to the growing interests in MHC ligand prediction, especially to the screening of effective neoepitopes for cancer vaccine development.

## Availability and requirements

**Project name:** MHCSeqNet

**Project home page:** https://github.com/cmbcu/MHCSeqNet

**Operating systems(s):** Platform independent

**Programming language:** Python 3

**Other requirements:** None

**License:** Apache 2.0

## Additional files

Additional file 1 Figure S1 – Illustration of the two alpha helices (blue and magenta) used to represent an MHC class I allele in our sequence-based model. The structure of HLA-B*35:01 from the Protein Data Bank entry 1A1N is shown here. Amino acid residue positions 50-84 and 140-179 correspond to the two alpha helices in this structure. Inclusion of the beta-sheet (yellow) decreased model performances. (PNG 210 kb)

Additional file 2 Table S1 – The MHC allele-specific AUC for each software tool evaluated on the binding affinity dataset. (TSV 8 kb)

Additional file 3 Table S2 – The cleaned peptide-MHC binding affinity dataset. (TSV 8645 kb)

Additional file 4 Figure S2 – Boxplots comparing the predicted binding probabilities between positive and negative classes. The orange horizontal lines indicate medians. Black boxes designate the 25^th^–75^th^ percentile regions. The whiskers indicate the non-outlier ranges which cover from Q1 - 1.5 * (Q3 - Q1) to Q3 + 1.5 * (Q3 - Q1), where Q1 and Q3 are the values at 25^th^ and 75^th^ percentiles, respectively. Black circles represent outliers. (PNG 56 kb)

Additional file 5 Figure S3 – Scatter plot comparing performances of sequence-based and one-hot models when tested on unseen MHC class I alleles not included in the training dataset. Each circle shows the AUC of one-hot and sequence-based model on each held-out allele. Colors indicate HLA types (A, B, or C). Circle sizes indicate the relative number of data points of the held-out allele. (PNG 79 kb)
