# Supplementary figures and images for: MHCSeqNet: a deep neural network model for universal MHC binding prediction
Source: BMC Bioinformatics. 2019 May 28;20:270. doi: 10.1186/s12859-019-2892-4 (PMC6540523; doi:10.1186/s12859-019-2892-4)

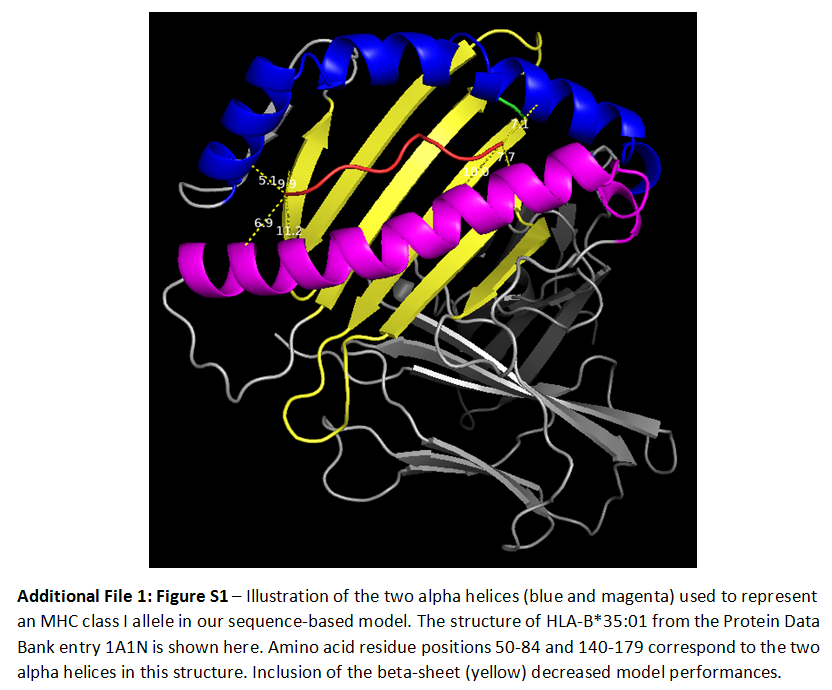

Supplement: Supplementary file 1 — Figure S1 – Illustration of the two alpha helices (blue and magenta) used to represent an MHC class I allele in our sequence-based model. The structure of HLA-B*35:01 from the Protein Data Bank entry 1A1N is shown here. Amino acid residue positions 50-84 and 140-179 correspond to the two alpha helices in this structure. Inclusion of the beta-sheet (yellow) decreased model performances. (PNG 210 kb) [file 12859_2019_2892_MOESM1_ESM.png]

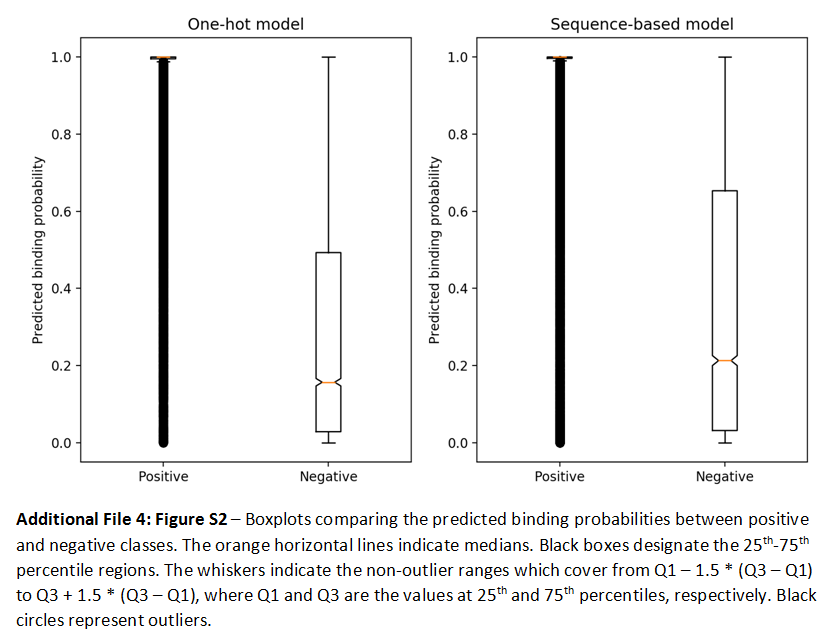

Supplement: Supplementary file 4 — Figure S2 – Boxplots comparing the predicted binding probabilities between positive and negative classes. The orange horizontal lines indicate medians. Black boxes designate the 25th–75th percentile regions. The whiskers indicate the non-outlier ranges which cover from Q1 - 1.5 * (Q3 - Q1) to Q3 + 1.5 * (Q3 - Q1), where Q1 and Q3 are the values at 25th and 75th percentiles, respectively. Black circles represent outliers. (PNG 56 kb) [file 12859_2019_2892_MOESM4_ESM.png]

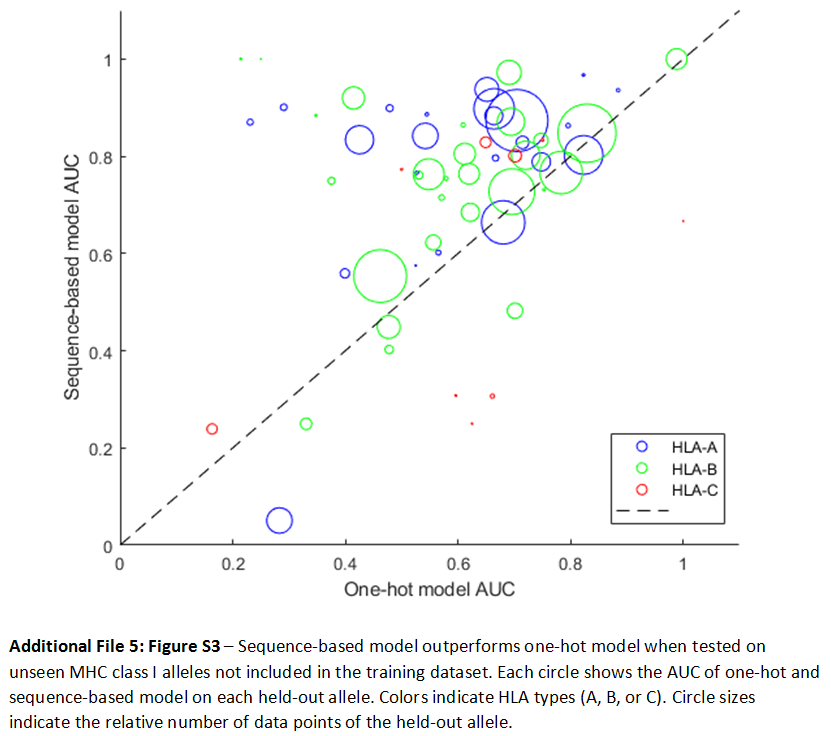

Supplement: Supplementary file 5 — Figure S3 – Scatter plot comparing performances of sequence-based and one-hot models when tested on unseen MHC class I alleles not included in the training dataset. Each circle shows the AUC of one-hot and sequence-based model on each held-out allele. Colors indicate HLA types (A, B, or C). Circle sizes indicate the relative number of data points of the held-out allele. (PNG 79 kb) [file 12859_2019_2892_MOESM5_ESM.png]
